# Social good reappraisal as a novel and effective emotion regulation strategy

**DOI:** 10.1371/journal.pone.0305756

**Published:** 2024-06-25

**Authors:** Nancy Tsai, Jade Hawkesworth, Jeff Dieffenbach, Dana Hua, Elena Eneva, John Gabrieli

**Affiliations:** 1 McGovern Institute for Brain Research and Department of Brain and Cognitive Sciences, Massachusetts Institute of Technology, Cambridge, Massachusetts, United States of America; 2 Department of Mechanical Engineering, Massachusetts Institute of Technology, Cambridge, Massachusetts, United States of America; 3 Accenture Labs, San Francisco, California, United States of America; 4 Harvard Graduate School of Education, Cambridge, Massachusetts, United States of America; 5 MIT Integrated Learning Iniative, Cambridge, Massachusetts, United States of America; University of Wyoming College of Health Sciences, UNITED STATES

## Abstract

Society asks individuals, such as front-line medical and emergency personnel or social media moderators, to help others under highly negative emotional circumstances, and those individuals need to regulate their emotions for their own well being. A well-studied form of emotion regulation is *reappraisal*, the use of cognitive processes used to reinterpret initial emotional responses to negative events. *Distancing* (pretending that a situation is distant in time or space) is well documented to be an effective form of emotion regulation, but it may not be applicable in social contexts where individuals must engage with distressing events to help others. Here, for the first time, we asked whether a novel reappraisal strategy focused on *Social Good*–imagining that an aversive event is also an opportunity to prevent harm to others–can be an effective form of reappraisal. In a pre-registered experiment, participants were randomly assigned to *Distancing* or *Social Good* conditions as they viewed neutral or highly aversive images and then reported their subjective emotional states with or without reappraisal. Both *Distancing* and *Social Good* reappraisals led to significantly less negative affect. *Distancing* yielded a stronger effect, but importantly, participants reported both *Distancing* and *Social Good* as equally easy to employ and both were effective across multiple demographic and personality characteristics, indicating the broad value of both as effective forms of reappraisal. Across both reappraisal conditions, effective reappraisal increased with age and positive affect. These findings indicate that *Social Good* is an effective reappraisal strategy and raise the possibility that it could be particularly valuable in contexts in which emotionally demanding tasks are completed on behalf of the good for other people.

## Introduction

The ability to cognitively modify how we experience emotions, known as emotion regulation, is a critical factor in mental health outcomes [e.g., 1, 2]. Among the most flexible and effective forms of emotion regulation that have been formally studied is cognitive reappraisal—altering the way in which a stimulus is interpreted so that one feels better. Much research has demonstrated reappraisal’s benefits for emotional, social, cognitive, and physiological outcomes for individuals across the lifespan [reviewed in 3] and as a result, reappraisal has played a key role in treatments used to remedy mood and anxiety disorders [e.g., 4]. In laboratory studies, for example, reappraisal has demonstrated success in decreasing both self-reports and physiological measures of negative affect and arousal [e.g., 5, 6]. In the brain, neuroimaging studies have shown that reappraisal is linked to increased activation in frontal regions associated with cognitive control and decreased activation in the amygdala that is associated with response to aversive events [7, 8, reviewed in 9].

One commonly used and well-researched reappraisal strategy is known as *Distancing* [10–12], which is increasing the psychological distance between oneself and a distressing cue. *Distancing* requires individuals to imagine a negative stimulus as happening far away, a long time ago, or from a third-person perspective. As a reappraisal strategy, *Distancing* has demonstrated reductions in self-reported negative affect [13–15] and biological indices of emotional arousal including blood pressure and amygdala activation [10, 16]. In a study of long-term use, *Distancing* was associated with diminished reports of stress in daily life [17] and, overall, has demonstrated promise as an effective form of emotion regulation inside and outside the laboratory [18]. However, successful implementation of *Distancing* and other forms of reappraisal may be challenging and variable as one might expect in everyday life. As many as fifty percent of participants in a laboratory study felt worse after attempting to reappraise as opposed to responding naturally [19, 20] and in an observational daily diary study, participants who were instructed to reappraise during their most stressful daily events rated their reappraisal success below average (i.e., not at all or only slightly successful) when facing negative life events [21].

The limitations of *Distancing* as a form of reappraisal suggest that it may not be applicable in some situations, particularly ones that are social in nature. Imagine fire fighters arriving to a burning home with a family with children trapped inside, an emergency room physician working to keep a patient alive, or a content moderator processing an endless stream of potentially psychologically harmful social media to prevent it from spreading to the wider public. These kinds of socially valuable actions demand engagement with, rather than distancing from, a distressing situation. A reappraisal strategy that increases engagement with the situation in a social manner may be a more relevant and therefore more effective form of reappraisal for individuals helping others under conditions that inherently invoke negative emotions.

Therefore, we asked for the first time whether actively thinking about the social good being done for others can serve as a method of reappraisal that reduces negative affect. Here, we developed a novel reappraisal strategy that focuses on an individual’s efforts towards *Social Good*–adopting an empowered mindset and recognizing that the negative situation presented is an opportunity to help others and prevent further harm. A number of studies have considered methods beyond *Distancing* as a form of cognitive reappraisal—indeed, positive reappraisal or “benefit finding” focuses on the positive aspects of a target situation and has demonstrated efficacy as a form of reappraisal [22, 23]. However, this is the first study to ask if consideration of *Social Good* as motivating engagement with aversive stimuli can reduce the negative emotional consequences of that necessary engagement.

In the present study, we examine the effectiveness of *Social Good*, *a novel and untested reappraisal strategy*, in relation to *Distancing*, *a well-researched and effective reappraisal strategy* commonly used in emotion regulation research. We hypothesized that 1) reappraisal irrespective of strategy-type would diminish negative affect (general effects of reappraisal), and 2) *Social Good* reappraisal would diminish negative affect to a similar degree as *Distancing* (differential effects of reappraisal type). Further, because of reports of variation among individuals on the effectiveness of using *Distancing* and other kinds of reappraisal [19, 20], we conducted exploratory analyses and asked participants about the ease of employing each kind of reappraisal, its potential applicability to everyday life, and whether the effectiveness of each kind of reappraisal varied in relation to demographic variables (age, gender, years of education, occupational status), personality variables (trait mindfulness, stress mindset, emotion regulation, resilience, strength of religious faith), or current well-being (negative or positive feelings over the past week). Exploring such individual differences and how they relate to reappraisal outcomes is a critical step in understanding for whom and in what circumstances these reappraisal strategies are differentially beneficial.

## Materials and methods

### Transparency and openness

We report how we determined our sample size, all data exclusions, all manipulations, all measures in the study, and we follow JARS [24]. We preregistered primary hypotheses, exploratory questions, methods, and data analytic approach before undertaking data collection on AsPredicted (https://aspredicted.org/XPP_P2Z). Data were analyzed using Stata version 13 [25]. Deviations in the pre-registered analytical approach occurred only to ensure the study was conducted in the most statistically conservative manner possible (i.e., using two-way repeated measures ANOVA vs. t-tests). This research was approved by the Committee on the Use of Humans as Experimental Subjects (IRB 2112000530A005: “Reappraisal of Social Distancing and Social Good in Adults”). The study was conducted virtually using Zoom video conferencing software and the behavioral task was programmed using PsychoPy2 experimental software [26] and hosted on Pavlovia, available online at https://pavlovia.org (accessed on August 25, 2022). Questionnaire responses were recorded using Qualtrics, available online at https://www.qualtrics.com (accessed on August 25, 2022). To assess the power of potential moderation effects which tend to have a small effect size [27], we used the G*Power software package [28] to calculate the ANOVA repeated measures within-between interaction statistical test (2 groups; 2 points of measurements) which indicated a total sample size of 200 (*n* = 100 for each reappraisal group) to achieve sufficient power (80%) to detect a small effect size (.10) at a < .05.

Given the remote nature of the study, all efforts to create a laboratory-like environment were made: participants were asked to be situated in a quiet room by themselves, use a laptop or desktop (i.e., phones or iPads/tablets were not allowed), and to keep their cameras on for the entire duration of the study to ensure they were focused on the task. After receiving the participant’s written informed consent, the researcher guided the participant through each of the task components, including the pre- and post-task questionnaires and the emotion regulation task, all described in detail below. At the end of the study, participants were provided with a debrief of the study. Data collection and participant recruitment occurred between January 6, 2022 to July 31, 2022.

### Design

We used a within- and between-subjects design and randomly assigned participants to one of two reappraisal conditions: *Distancing* or *Social Good*. All participants regardless of condition assignment completed (1) pre- and post-task questionnaires, (2) training in reappraisal (*Distancing* or *Social Good*) and control instructions (“look”; no regulation) (see below for task instructions), and (3) an image-based regulation task using images from the *International Affective Picture System* [IAPS; 29]. Sixty highly aversive images were selected from the IAPS (valence: M = 2.05, S.D. = 0.32; arousal: M = 6.12, S.D. = 0.66) along with 30 neutral images from the IAPS (valence: M = 5.22, S.D. = 0.41; arousal: M = 3.54, S.D. = 0.84); each image was used only in one trial. Neutral images were only paired with the “Look” instruction to serve as a baseline whereas negative images were used with either “Look” or “Reframe” instructions and matched for valence (Look: M = 2.05, SD = 0.28; Reframe: M = 2.06, SD = 0.36) and arousal (Look: M = 6.01, SD = 0.76; Reframe: M = 6.21, SD = 0.54). Negative images included a wide variety of emotional scenes (e.g., bodily injury, people expressing anger, assault) and the two lists were matched on image content.

#### Task instructions

Participants received the following set of instructions below. All participants performed the *Look* task with neutral scenes and half of the negative scenes. Participants in the *Distancing* group performed *Distancing* as the method of reappraisal for half of the negative scenes. Participants in the *Social Good* group performed social good as the method of reappraisal for half of the negative scenes.

*Look*. “One instruction will tell you to Look at the image. When you see this instruction, we want you to attend to the image and respond naturally. Allow yourself to experience any emotions that may or may not be evoked in you. The key is to look at the image and allow yourself to feel the emotions that you experience while looking at the events depicted.”

*Distancing*. “While performing the task, try to adopt a neutral and objective attitude by thinking about your performance from a third-person perspective that is detached from the outcome of the task. In other words, feel as though you are indifferent and emotionally detached from your performance. For example, while looking at an image, imagine that it is a screenshot from a movie. Try and detach yourself from the task itself.” Summarized points were presented at the bottom of the instructional screen, “1) adopt a neutral and objective attitude toward your performance; 2) think about it from a third person perspective; 3) detach yourself from any emotional experience or evaluation of your performance. The key is to change, or reframe, the way you think about the image so that you feel less emotion.”

*Social good*. “While looking at the image, try to adopt a powerful attitude by thinking about your performance as a service of social good. In other words, feel as though you are saving people from harm or preventing similar tragedy from happening to others. Remember that you’re taking on a powerful role with your performance and the task ahead.” Summarized points were presented at the bottom of the instructional screen, “1) adopt a powerful attitude toward your performance; 2) think about it from a Social Good perspective; 3) think about the social good of protecting people from harm. The key is to change, or reframe, the way you think about the image so that you feel less emotion.”

#### Task training

Each session consisted of two phases each lasting approximately 30 minutes: (1) standardized reappraisal/control instructions presented by the experimenter, and (2) task completion by the participant. Each participant received a REFRAME instruction for either the *Social Good* or *Distancing* reappraisal technique, as well as the LOOK instruction. Aside from description of either technique, each participant underwent identical training. During training participants were assessed on their understanding of the task through a 3-question comprehension quiz.

#### Emotion regulation tasks

Participants were presented two tasks: one of the two reappraisal tasks (“Reframe”; *Distancing* or *Social Good*, pending condition assignment) and one control task (“Look”) while viewing negative or neutral IAPS images. Tasks were presented as trials with 30 trials in a round and participants completed three total rounds, with brief self-paced breaks in between each round to prevent fatigue. Trials were divided into three conditions: (i) look neutral, (ii) look negative, (iii) reframe negative. In total, participants viewed 30 neutral images and 60 negative images, each image viewed only once and presented in the same pseudo-randomized order for each participant. We used PsychoPy’s built-in loop randomizer to select trials at random from a list of trials grouped by type such that the randomizer was unlikely to choose trials of the same type sequentially.

**Fig 1:**
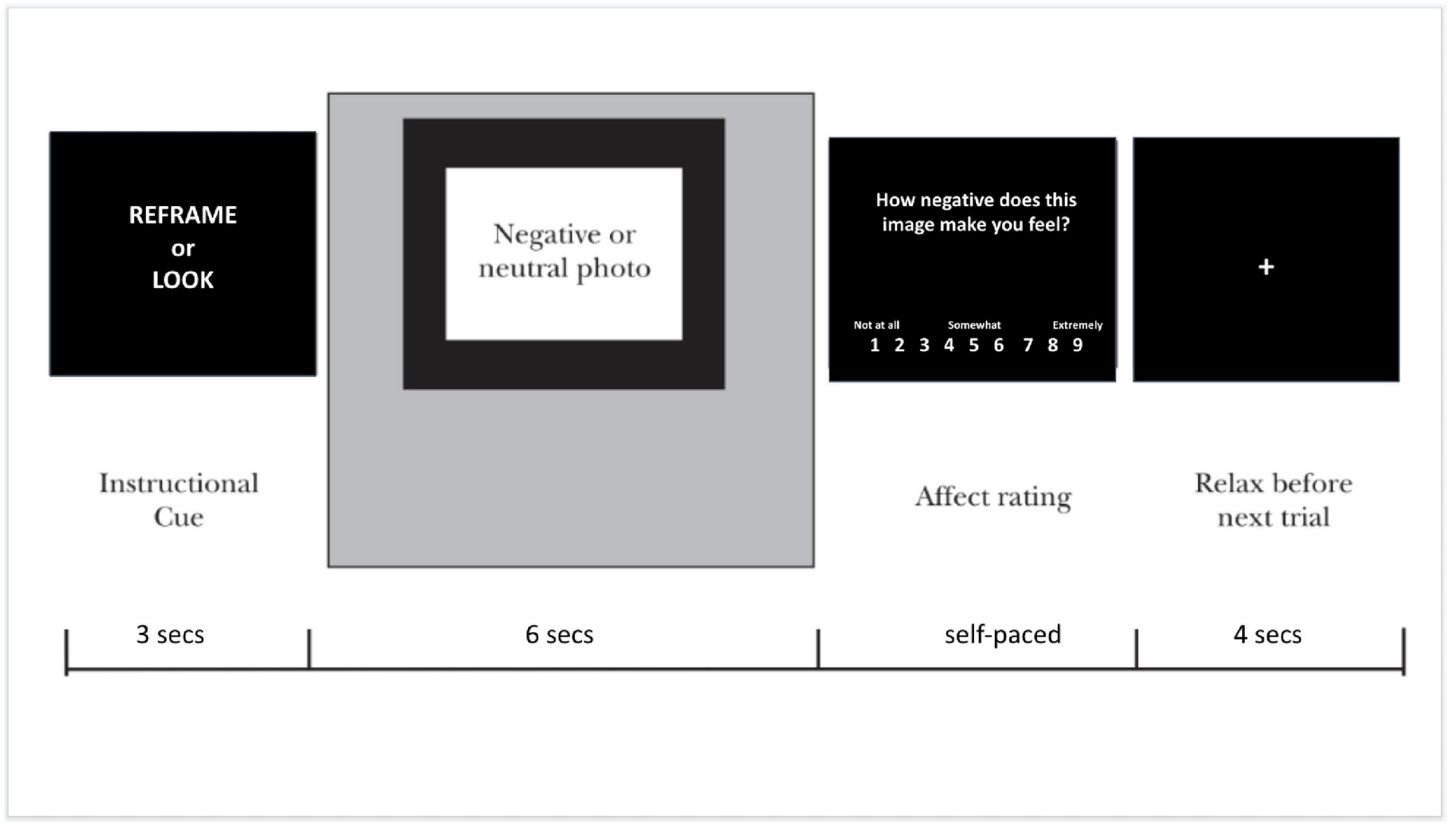
Timeline for events on each trial. An initial cue instructs participants to Reframe or Look, which is followed by a photo presentation period during which participants follow the instruction. Participants then provide a rating of their current negative affect and finally hve a moment to relax before the onset off the next trial.

At the beginning of each round, a slide with bulleted points summarized the Look and Reframe strategy. Each trial consisted of (1) a trial-specific instruction appearing for three seconds (Look or Reframe); (2) a neutral or negative IAPS image appearing for six seconds; and (3) a self-paced rating period that asked participants, “How negative does this image make you feel?” with a 9-point scale (1 = Not at all, 9 = Extremely) below it. There was a five second countdown timer at the corner of the screen that reminded the participant to respond within five seconds. If the countdown went to zero, it would prompt the participant to “Please respond now!” but would not force a rating or change screen. After the rating, a fixation cross was shown for four seconds (Fig 1). Once all 30 trials in a round were presented, the participants were offered a brief break. At the end of the session, participants were debriefed, thanked, and paid $20 for their participation.

#### Self-report measures

The following questionnaires were administered pre-task, following study overview but prior to the administration of the reappraisal task. The pre-task questionnaire began with demographic questions including age, gender, race, highest level of education completed (i.e., some high school; completed high school; some college; some graduate studies; completed graduate studies), employment status (i.e., employed and working 40 or more hours per week; employed and working 1–39 hours per week; not employed and looking for work; not employed and not looking for work; retired; disabled and not able to work), and occupation (19 occupations types were listed such as “sales occupations”) before moving on to multiple standardized measures related to affect and affect processing. The following personality (e.g., emotion regulation, stress mindset, emotion regulation, resilience, strength of religious faith) and current well-being (e.g., affect and stress) variables were used to explore individual baseline differences in these areas as they relate to reappraisal effectiveness.

The **Emotional Regulation Questionnaire** [ERQ; 30] assesses subjective emotional control with 10-items using two strategies, cognitive reappraisal (Cronbach’s α = 0.827) and expressive suppression (Cronbach’s α = 0.761). Each question was assessed using a 5-point scale (1 = Strongly Disagree, 5 = Strongly Agree) and looked at two aspects of emotion regulation, using items such as “When I want to feel happier, I think about something different” for cognitive reappraisal and “I keep my feelings to myself” to assess expressive suppression.

The **Stress Mindset Measure** [SMM; 31] assesses the extent to which a participant perceives the consequences of stress in a positive light with 8 items. Each question was assessed on a 5-point rating scale (1 = Strongly Disagree, 5 = Strongly Agree). Items such as “Experiencing stress facilitates my learning and growth” helped to determine a “stress-is-enhancing” or “stress-is-debilitating” mindset. Consistent with many of the questionnaires used, the SMM has high levels of internal consistency concerning its total score and subscores (α = 0.80–0.86).

The **Mindful Attention Awareness Scale** [MAAS; 32] measures an individual’s awareness and attentiveness of events taking place in the present with 14 items. Statements such as “I tend to walk quickly where I’m going without paying attention along the way” are rated on a 5-point scale (1 = Almost Never, 5 = Almost Always). Individuals with a high average rating can be characterized as having a higher dispositional mindfulness, which is related to greater emotional control and self-regulation. MAAS shows excellent internal consistency (α = 0.89–0.93).

The **Positive and Negative Affective Schedule** [PANAS; 33] assesses emotional well-being over the past week using 10 items. Each item was rated on a 5-point scale (1 = Never, 5 = Always) and related to assessing an individual’s perception of feeling certain emotions, 5 items being of positive affect (ex. inspired, determined) and 5 items of negative affect (ex. ashamed, nervous). PANAS is widely used for emotional measures, with a moderately good Cronbach’s alpha on the positive affect (α = 0.86–0.90) and negative affect (α = 0.84–0.87) subscales.

The **Brief Resilience Scale** [BRS; 34] has 6 items concerning stress and recovery, such as “I tend to bound back quickly after hard times” and “It does not take me long to recover from a stressful event.” This measurement helps to assess the impact of stressful events on participants, to better understand the effectiveness of reappraisal implementation. BRS has a sufficient internal consistency (α = 0.78), with an advisory note to look for inconsistent responses from respondents that may affect its validity.

The **Santa Clara Strength of Religious Faith questionnaire** [SCSORF; 35] examined perceptions of faith and religion, which was collected to evaluate its relation to reappraisal effectiveness. The instrument consisted of 10 items on a 4-point scale (1 = Strongly Disagree, 4 = Strongly Agree). The questionnaire asks about religious and spiritual faith and its impact on individual behaviors. SCSORF shows adequate internal consistency (α = 0.89).

The **State-Trait Anxiety Inventory** [STAI; 36] is a 7-item questionnaire to measure the anxiety levels of participants. The items were assessed on a 5-point scale (1 = Not at all, 5 = Extremely), and exhibited high levels of internal consistency on both the state (α = 0.93) and trait (α = 0.92) subscales. The seven items were used in the pre-task questionnaire to measure baseline anxiety to assess any differences before viewing the IAPS images.

Three exploratory questions were administered at post-task to assess the participants’ perception of the effectiveness of reappraisal strategies. The questions asked about the Look and Reframe strategy (“How effectively did you feel you were able to implement the Look strategy?” and “How effectively did you feel you were able to implement the Distancing [or Social Good] strategy?”), and the perceived applicability of the Reframe strategy in everyday life (“To what degree do you think the strategies learned in this study are applicable to your daily life?”) and answered on a 100-point Likert scale (0: Not well at all; 100: Very well).

### Data analysis

Behavioral and questionnaire data were analyzed using Stata software [25]. Our analytic approach described below follows our AsPredicted registered plan. The primary aim of our study was to examine whether (1) reappraisal strategies effectively mitigate the self-report of negative affect after viewing negative IAPS images (General effects of reappraisal: Hypothesis (1), and (2) how *Social Good* as a reappraisal strategy compared to Distancing reappraisal (Differential effects of reappraisal type: Hypothesis 2)). Data and materials will be made available to other researchers upon request and will be made posted on a public site following publication.

## Results

### Participants

Participants were 259 English-speaking adults ages 18–55 who were recruited from the service Prolific. Beyond the age range there were no exclusion criteria. They were randomly assigned to either the *Distancing* or *Social Good* group. Participants (*n = 55)* were excluded for the following reasons: not paying attention to the task which impaired their ability to sufficiently complete tasks (*n = 12*), failing to understand instructions and/or complete all pieces of the study (*n = 18*), technical issues (*n = 17*), or voluntary withdrawal from the study before beginning (*n = 8*). Data collection was halted once we reached our target sample size. Thus, reported results reflect data from 204 participants (*n* = *104* in the Distancing condition [mean age = 34.08 years, S.D. = 9.08, 53 female]; *n = 100* in the Social Good [mean age = 33.26 years, S.D. = 8.53, 50 female]). Each participant was categorized by level of education (some high school; completed high schools; some college; completed college; some graduate studies; completed graduate studies) and occupational status (Employed, working 40 or more hours per week; Employed, working 1–39 hours per week; Not employed, looking for work; Not employed, not looking for work; Retired; Disabled, not able to work). Participants were compensated $20 for their time.

### Participant characteristics

There were no statistically significant demographic, personality, or emotional well-being differences between the *Distancing* and *Social Good* groups at baseline. The two groups did not differ by age, gender composition, race composition, level of education completed, employment status, or occupation type (Table 1). Furthermore, the groups did not differ at baseline in their self-reported emotion regulation (t(202) = 0.31, *p* = .75, *d* = -.04), stress mindset (t(202) = 1.30, *p* = .19, *d* = .18), dispositional mindfulness (t(202) = 0.13, *p* = .89, *d* = .01), state-trait anxiety (t(202) = 0.36, *p* = .71, *d* = .05), positive affect (t(202) = .71, *p* = .47, d =.-.1), negative affect (t(202) = 1.47, *p* = .14, *d* = -.2), resilience (t(202) = 0.43, *p* = .66, *d* = -.06), or religiosity (t(202) = 0.68, *p* = .49, *d* = .09).

**Table 1:** Demographic characteristics for participants as a function of reappraisal group.

Variable	*Distancing* (*n* = 104)	*Social Good* (*n* = 100)	Difference between samples	
			Test statistic	*p*
Age (years)	*M* = 34.1 (*SD* = 9.03)	*M* = 33.3(*SD* = 8.53)	*t*(202) = 0.66	.50
Gender			*X*^2^ (2, *N* = 204) = 0.02	.99
Female	51%	50%		
Male	44.2%	45%		
Non-binary	0.0%	0.0%		
Other	4.8%	5%		
Race (%)			*X*^2^ (13, *N* = 204) = 15.2	.30
American Indian/Alaskan Native	0.0%	0.0%		
Asian	11.5%	8%		
Black	9.6%	14%		
Native Hawaiian / Pacific Islander	0.0%	0.0%		
White	66.2%	62%		
Hispanic or Latino	5.8%	5%		
Multiple Race	5.9%	7%		
Decline to state	1.0%	4%		
Education Level (%)			*X*^2^ (5, *N* = 204) = 2.18	.82
Some high school	0.0%	1.0%		
Completed high school	4.8%	6.0%		
Some college	20.2%	20.0%		
Completed college	49.0%	44.0%		
Some graduate studies	5.8%	9.0%		
Completed graduate studies	20.2%	20.0%		
Employment Status (%)			*X*^2^ (3, *N* = 204) = 1.68	.64
Employed, 40+ hrs/week	61.5%	65.0%		
Employed, 1–39 hrs/week	26.0%	23.0%		
Not employed, looking	11.5%	9.0%		
Not employed, not looking	1.0%	3.0%		
Retired	0.0%	0.0%		
Disabled, not able to work	0.0%	0.0%		

To examine the effect of trial type (within-subjects) and reappraisal condition (between-subjects) on self-reported negativity ratings, a two-way repeated measures ANOVA revealed a statistically significant main effect for trial type (F(2, 404) = 1135.2, *p* < .001, ηp^2^ = .849), reappraisal condition (F(1, 202) = 9.00, *p* = .003, ηp^2^ = .043), and an interaction effect between trial type and reappraisal condition (F(2, 404) = 16.7, p < .001, ηp^2^ = .076). This indicates that the self-reported negativity ratings differed across trial types and reappraisal conditions, and that the negativity ratings also differed across different trial types (Look-Neutral, Look-Negative, Reappraise-Negative) for *Distancing* and *Social Good* groups. This was followed by Bonferroni-adjusted pairwise comparisons reported below to examine differences across trial types and reappraisal condition. The data satisfied the assumptions of normality, homogeneity of variance, and sphericity.

#### Hypothesis 1 (within-subject results): General effects of trial type and reappraisal

Participants reported feeling significantly more negative affect for the Look-Negative trials than the Look-Neutral Trials in both reappraisal conditions (*Distancing M*_Diff_ = 4.64, *SE* = .14, *t*(202) = 32.80, *p* < .001, *d* = 3.65; *Social Good M*_Diff_ = 4.78, *SE* = .14, *t*(202) = 33.139, *p* < .001, *d* = 3.70). Participants also reported feeling significantly less negative affect after reappraisal of negative scenes than after Look-Negative trials in both reappraisal conditions (*Distancing M*_Diff_ = 2.24, *SE* = .13, *t*(202) = 16.57, *p* < .001, *d* = -1.62; *Social Good M*_Diff_ = 1.32, *SE* = .13, *t*(202) = 9.55, *p* < .001, *d* = —.95). (Table 2). These analyses confirm that self-reported negativity was lowest in the Look-Neutral trials, highest in the Look-Negative trials, and that reappraising negative images, irrespective of reappraisal type, was effective at lowering self-reported negativity.

**Table 2:** Descriptive data of self-reported negativity ratings by trial as a function of reappraisal group.

Reappraisal Condition	Trial Condition			
		N	Mean	SD
**Social Good**				
	Look-Neutral	100	1.25	0.34
	Look-Negative	100	6.03	1.42
	Reappraise-Negative	100	4.72	1.61
	Total	300	4.00	2.38
**Distancing**				
	Look-Neutral	104	1.25	0.31
	Look-Negative	104	5.89	1.61
	Reappraise-Negative	104	3.65	1.48
	Total	312	3.60	2.28
**Total**				
	Look-Neutral	204	1.25	0.33
	Look-Negative	204	5.96	1.52
	Reappraise-Negative	204	4.17	1.63
	Total	612	3.80	2.34

#### Hypothesis 2 (between-subject results): Differential effects of reappraisal type

**Fig 2:**
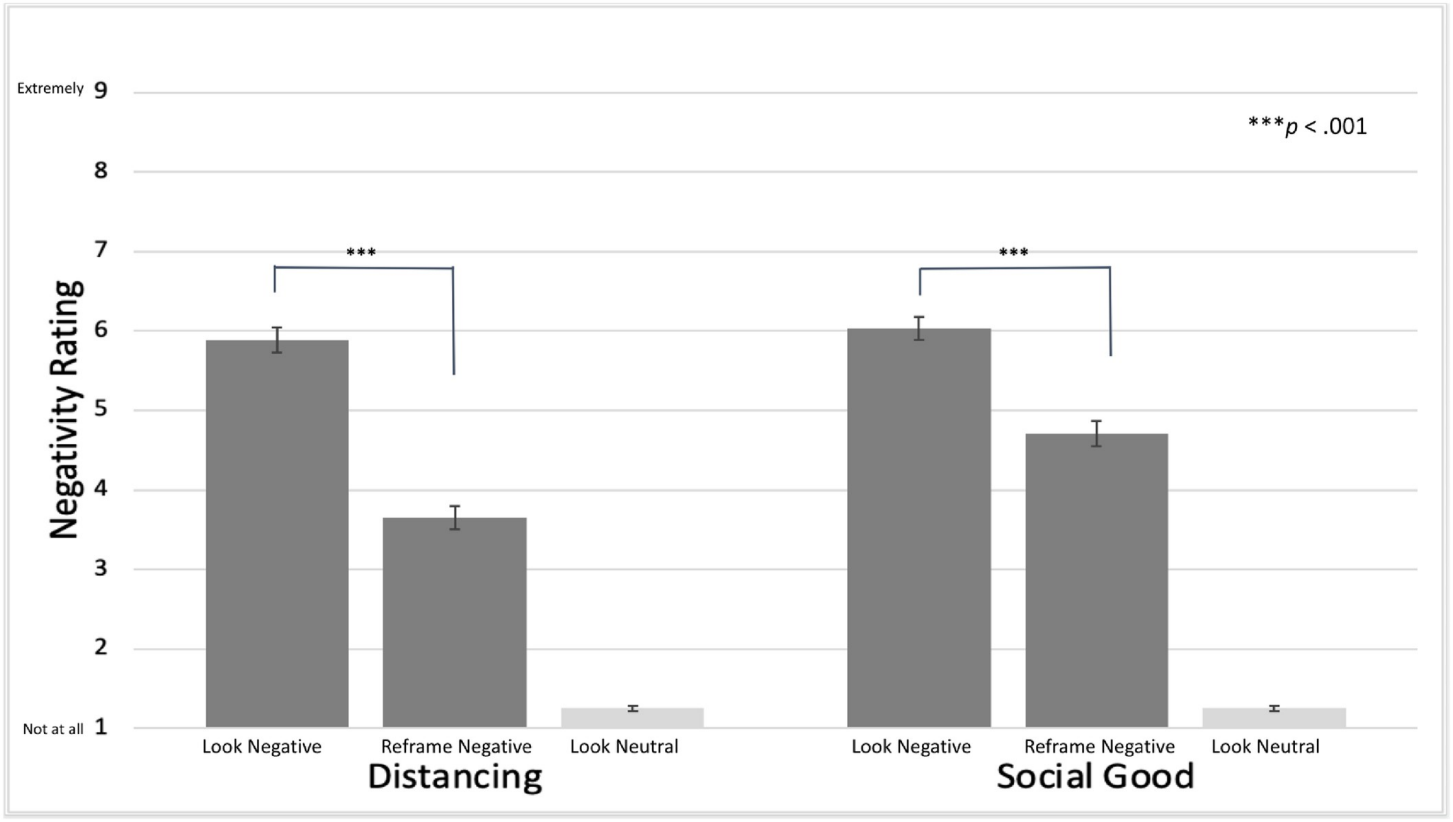
Average negative ratings made during trials by trial type and reappraisal group. Negative ratings were highest during the Look-Negative trials (*Distancing*: *M* = 5.89, *SD* = 1.61; *Social Good*: *M* = 6.03, *SD* = 1.42) and lowest for Look-Neutral (*Distancing*: *M* = 1.25, *SD* = 0.31; *Social Good*: *M* = 1.25, *SD* = 0.33). When partiicpants were asked to reframe when presented negative images, reframing significantly lowered the negativity ratings for both *Distancing and Social Good* groups, although *Distancing* lowered negativity to a greater degree (*Distancing*: *M* = 3.54, *SD* = 1.47; *Social Good*: *M* = 4.72, *SD* = 1.61).

We examined the differential effects of the two kinds of reappraisal in the *between-subject* analysis comparing effects of the *Distancing* and *Social Good* groups on negativity ratings. Participants in the *Distancing* and *Social Good* groups did not differ significantly in their ratings for the Look-Neutral trials (*M*_Diff_ = 0.00, *SE* = .04, *t*(202) = 0.07, *p* = .94, *d* = -.01) or Look-Negative trials (*M*_Diff_ = -0.14, *SE* = .21, *t*(202) = 0.67, *p* = .50, *d* = -.09). Ratings of negative affect after reappraisal differed between groups, with the *Distancing* group reporting lower negative ratings than the *Social Good* group (*M*_Diff_ = 1.06, *SE* = .21, *t*(202) = 4.92, *p* < .001, *d* = -.69) (Fig 2).

Also, participants in the two reappraisal groups did not differ significantly in their reports about how well they could implement *Social Good* versus *Distancing* reappraisals (*Social Good*: *M* = 70.2, *SD* = 20.4; *Distancing*: *M* = 70.8, *SD* = 21.6, t(202) = 0.84, *p* = .40, *d* = -.02). They also did not significantly differ in their judgments about the potential applicability of *Social Good* versus *Distancing* reappraisals in everyday life (*Social Good M* = 69.0, *SD* = 24; *Distancing M* = 65.8, *SD* = 26.4; t(202) = 0.924, *p* = .357, *d* = -.13).

In the Preregistration we stated that we would analyze reappraisal findings in a series of *t* tests. The repeated measures ANOVAs reported above are a more conservative approach to analying the findings. However, the individual *t* tests yielded identical findings of no differences between groups for Look-Neutral (*p* = .93) and Look-Negative trials (*p* = .50), lower ratings for Reappraise-Negative trials for both reappraisal conditions (*p*s < .001), and a greater decrease in negative affect when reappraising negative images (Look-Negative minus Reappraise-Negative) for those in the *Distancing* group compared to the *Social Good* group (*p* < .001).

### Individual differences

We also conducted exploratory analyses to examine how individual attributes at baseline related to reappraisal efficacy or *difference* in self-reported negative affect between negative trial types (Look-Negative minus Reappraisal-Negative). Among demographic variables of interest, there were no significant differences in relation to sex or race but increasing age correlated with greater reappraisal efficacy in both conditions (*Distancing r* = .23, *p* < .05; *Social Good r* = .32, *p* < .001) and greater educational attainment correlated with greater efficacy in *Social Good* reappraisal (*r* = .23, *p* < .02). Among the affective measures, more positive current emotional well-being (PANAS) correlated significantly with greater reappraisal efficacy in both conditions (*Distancing r* = .32, *p* < .001; *Social Good r* = .25, *p* < .01). The differences in the correlations for age and positive affect were not statistically significant across the two regulation conditions (age, *p* = 0.49; positive affect *p* = 0.59). Among the multiple personality measures, more positive attitude towards stress (SMM) correlated with more successful *Social Good* reappraisal (*r* = .19, *p* = .05) and more dispositional mindfulness (MAAS) correlated with less successful *Distancing* reappraisal (*r* = -.20, *p* = .03) (Table 3).

**Table 3:** Pearson’s correlations for individual differences.

	1	2	3	4	5	6	7	8	9	10	11	12	13	14
1. Dis_Ch	-													
2. SG_Ch	-	-												
3. ERQ	-.10	0.11	-											
4. SMM	.01	**.19** *	.12	-										
5. MAAS	**-.20** *	-.06	-.03	-.13	-									
6. STAI	-.16	-.07	-.14*	-.22***	.39***	-								
7. PA	**.32** ***	**.25** **	.24***	.23***	-.30***	-.33***	-							
8. NA	-.12	.00	-.13	-.25***	.45***	.50***	-.24***	-						
9. BRS	.07	.10	.30***	.34***	-.28***	-.45***	.47***	-.47***	-					
10. SCSORF	.17	.16	.05	.04	-.08	.02	.12	.05	-.13	-				
11. Age	**.23** *	**.32** ***	-.02	.10	-.10	-.01	.18**	.00	.09	.10	-			
12. Gender	-.001	-.06	.02	-.10	.02	.00	-.06	-.09	-.03	-.13	-.21**	-		
13. Edu	.05	**.23** *	.-07	.13*	.02	.00	.11	-.05	.10	.04	.26***	-.17*	-	
14. Race	.00	.08	-.05	.01	.09	.05	-.01	.02	.09	-.01	.07	.17	-.11	-

Dis_Ch = difference in self-reported negative affect between negative trial types and the average self-reported negative affect for the Look-Neutral trials for *Distancing* condition; SG_Ch = difference in self-reported negative affect between negative trial types and the average self-reported negative affect for the Look-Neutral trials for *Social Good* condition; ERQ = Emotion Regulation Questionnaire; SMM = Stress Mindset Measure; MAAS = Mindful Attention Awareness Scale; STAI = State-Trait Anxiety Inventory; PA = Positive Affect subscale of the Positive and Negative Affective Schedule; NA = Negative Affect subscale of the Positive and Negative Affective Schedule; BRS = Brief Resilience Scale; SCSORF = Santa Clara Strength of Religious Faith questionnaire; Race and Gender are treated as ordinal variables here.

*p < .05,

**p < .01,

***p < .001;

values in bold reflect individual differences that significantly or tended to significantly correlate with reappraisal efficacy

## Discussion

The present research shows, for the first time, that consideration of the social good motivating engagement with aversive events constitutes an effective form of reappraisal and emotion regulation. We originally hypothesized that 1) both *Distancing* and *Social Good* would decrease self-reported negative affect and that 2) *Social Good* reappraisal would decrease self-reported negative affect as much as or more than *Distancing*. Although *Social Good* reappraisal was effective in reducing negative affect, *Distancing* reappraisal was more effective. The effectiveness of *Distancing* for reappraisal replicates multiple prior behavioral [e.g., 10–12] and neuroimaging [e.g., 37–39] studies. Irrespective of the relative efficacy to *Distancing*, the present study unveils *Social Good* as an effective form of reappraisal. *Social Good* as an alternate form of reappraisal may potentially be helpful in circumstances in which individuals must actively engage in aversive conditions in order to aid others and when *Distancing* is not feasible.

The effectiveness of *Social Good* aligns with prior research on positive reappraisals [e.g., 40]. While positive reappraisal focuses on “silver linings” and finding positive meaning from a target situation, *Social Good* reappraisal instead focuses on the *individual* as opposed to the *situation* in creating positive meaning in a negative situation (e.g., “*I* can alert others so no one else will come in harm’s way”) [41]. Examining the underlying mechanism of this novel form of reappraisal was beyond the scope of the current study, but future research would benefit from assessing whether *Social Good* and positive reappraisal share mechanisms of positive psychological processes that can mitigate the emotional consequences of negative experiences. Indeed, the correlation between positive well-being at the outset of the present experiment and greater reappraisal efficacy further supports the relation between positive emotional mechanisms and how well reappraisal can mitigate negative affect in response to aversive experiences.

There were similarities and differences between the *Distancing* and *Social Good* reappraisal outcomes that indicated for whom the strategies would be more or less effective. Both participants assigned to *Distancing* and *Social Good* reappraisal groups reported that the reappraisals were easy to implement during the experimental task and in everyday life. While both forms of reappraisal were broadly beneficial, they were also similar in that both were more effective with increasing age and more positive well being as discussed below.

Increasing age from 18 to 55 years of age was associated with more effective reappraisal for both *Social Good* and *Distancing*. These findings align with evidence suggesting that emotion regulation abilities improve as people age. In observational studies, older adults have reported being better at controlling their emotions relative to younger adults [42] and having greater decreases in negative emotion when asked to focus attention away from an upsetting film clip to a positive autobiographical memory [43]. There is also evidence, however, that older adults relative to younger adults are less successful in using cognitive reappraisal to decrease unpleasant emotion [44], although the older adults in that prior study were 5–15 years older than the age range included in the current study. The effect of age on emotion regulation may depend on the type of reappraisal involved. For example, older adults were less successful than younger adults when using detached reappraisal but more successful when using positive reappraisal [45]. Although we found a positive relation between increasing age and reappraisal efficacy, it is possible that different mechanisms may contribute to the two kinds of reappraisal, similar to the differences in neural mechanisms found underlying self- and situation-focused reappraisals [46].

More positive emotional well-being at baseline (but not negative emotions) was also related to greater reappraisal efficacy for both *Distancing* and *Social Good*. Although emotion regulation is assumed to improve everyday affect, only a few studies have directly examined this link between emotion regulation and positive well-being. The specific relations between positive well-being as measured by the PANAS [33] and reappraisal efficacy found in the current study are similar to another study of reappraisal that reported greater reappraisal efficacy for individuals with more positive well-being [47 and also 48 with a different measure of well-being]. Another study examining Japanese undergraduate students found that self-reported positive affect was positively related to cognitive reappraisal [49]. When examining the link between age and psychological well-being, researchers have found that this relationship was driven by the mediating link between reappraisal and positive affect [50, 51]. Unlike measures of traits, which are by definition less malleable over short periods, it is possible to enhance positive well-being over short periods. These findings suggest that enhancing positive well-being may amplify reappraisal benefits and thus more effectively diminish negative affect after aversive experiences.

There were few relations between the multiple trait measures we administered and reappraisal efficacy. Although any of the measured traits could have plausibly impacted reappraisal efficacy, it was surprising that self-reported emotion regulation [Emotional Regulation Questionnaire (ERQ); 30], an assessment of habitual strategy use, was unrelated to reappraisal efficacy. We may expect habitual emotion regulation to be related to reappraisal efficacy, but other studies have also found self-report measures of emotion regulation unrelated to reappraisal outcomes [10, 47, 52, 53]. Our use of a single-item measure of self-reported negativity as the dependent variable of reappraisal efficacy, the standard approach in many studies of reappraisal, may not be sufficiently sensitive in capturing these differences and is also a notable limitation.

In contrast to the participant characteristics of age and positive affect that were similarly related to greater *Distancing* and *Social Good* reappraisal efficacy, three other characteristics were related to only one or the other kind of reappraisal. Greater *Social Good* reappraisal efficacy was associated with (1) a stress mindset that was more likely to view stress as enhancing rather than debilitating, and (2) a higher education level. In contrast, lesser *Distancing* reappraisal efficacy was associated, unexpectedly, with greater dispositional mindfulness. The trending association between increased *Social Good* reappraisal efficacy and a stronger stress-is-enhancing mindset may highlight psychological mechanisms such as cognitive malleability that underly both reappraisal and stress mindset [54]. These exploratory examinations between individual characteristics and reappraisal efficacy may merit further investigation in future studies.

Several limitations and opportunities for future studies may be noted. Although *Distancing* and *Social Good* reappraisal shared some similarities and both significantly reduced negative affect, the reduction was greater after *Distancing*. To understand why this might be, the influence of situational context may be considered. Research findings indicate that the effectiveness of emotion regulation strategies depend on the specific context in which they are used [55, 56]. By designing our on-line study to mimic an in-person laboratory experience as closely as possible, the virtual environment and task may have been a limitation in that participants may have found *Distancing* as better matched as a strategy than *Social Good* in this unique context. That is, while viewing a negative image (e.g., a violent car crash) on a computer screen, it may be easier to imagine that the scene happened many years ago or was from a movie clip than imagine oneself imbedded in the actual scene as an empowered civilian calling for help. It is possible that when a person is engaged with aversive experiences off screen while actually needing to help others in person, *Social Good* would offer more powerful reappraisal than when a person is pretending there is social good in an on-line research experiment. However, without experimentally testing these reappraisal strategies in such active social environments, the possibility that *Social Good* would be more effective than *Distancing* is a hypothesis. Moreover, the types of images used in the study varied which may also influence how easily *Social Good* is implemented. For example, *Social Good* may be easier to employ for an image of a house on fire than an image of a grieving visitor at a graveyard; future studies could systematically control image types. Similarly, participants in the current study were sampled from a general rather than a specialized population limiting whether the present findings translate to specific subpopulations (e.g., first responders). Overall, examining *Social Good* in our current study highlighted positive and promising findings, but future studies would benefit from examining *Social Good* reappraisal outside the laboratory with specialized populations who perform social good in their jobs.

An important goal of emotion regulation research is not only understanding the psychological mechanisms of such regulation, but to empower individuals with contextually appropriate reappraisal strategies to overcome the negative emotions that are driven by aversive experiences. Given the strong positive relationship between reappraisal and psychological health [30, 57] and the importance of emotion regulation choice [58], researchers must ask which types of strategies are effective, in what contexts, and for whom. Successful emotion regulation requires *flexibility*—strategies to be adapted to changing situational demands and goals [59]. *Distancing* as a form of reappraisal has proven to be reliably effective as a reappraisal strategy, but it may be unemployable in social situations that demand engagement with negative emotions. The present study unveiled the potential of *Social Distancing* as an effective form of reappraisal. Future translational studies conducted in more socially realistic contexts and with more relevant populations may reveal whether *Social Good* reappraisal can better support the well-being of those tasked with social challenges in an effort to help others.
